# DNA-Binding Interaction Studies of Microwave Assisted Synthesized Sulfonamide Substituted 8-Hydroxyquinoline Derivatives

**DOI:** 10.3797/scipharm.1102-16

**Published:** 2011-05-01

**Authors:** Ritu B. Dixit, Tarosh S. Patel, Satish F. Vanparia, Anju P. Kunjadiya, Harish R. Keharia, Bharat C. Dixit

**Affiliations:** 1 Ashok and Rita Patel Institute of Integrated Study and Research in Biotechnology and Allied Sciences, New Vallabh Vidyanagar-388 121, Gujarat, India; 2 Department of Chemistry, V. P. & R. P. T. P. Science College, Vallabh Vidyanagar-388 120, Gujarat, India; 3 B & R Doshi School of Bio-Sciences, Sardar Patel University, Vallabh Vidyanagar-388 120, Gujarat, India

**Keywords:** Plasmid DNA, Calf Thymus DNA, Sulfonamides, 8-Hydroxyquinoline, Transition Metal Complexes

## Abstract

Sulfonamide substituted 8-hydroxyquinoline derivatives were prepared using a microwave synthesizer. The interaction of sulfonamide substituted 8-hydroxyquinoline derivatives and their transition metal complexes with Plasmid (pUC 19) DNA and Calf Thymus DNA were investigated by UV spectroscopic studies and gel electrophoresis measurements. The interaction between ligand/metal complexes and DNA was carried out by increasing the concentration of DNA from 0 to 12 μl in UV spectroscopic study, while the concentration of DNA in gel electrophoresis remained constant at 10 μl. These studies supported the fact that, the complex binds to DNA by intercalation via ligand into the base pairs of DNA. The relative binding efficacy of the complexes to DNA was much higher than the binding efficacy of ligands, especially the complex of Cu-AHQMBSH had the highest binding ability to DNA. The mobility of the bands decreased as the concentration of the complex was increased, indicating that there was increase in the interaction between the metal ion and DNA. Complexes of AHQMBSH were excellent for DNA binding as compared to HQMABS.

## Introduction

DNA is often referred as the molecule of heredity, as it is responsible for the genetic propagation of all traits [1–3]. Over the past decades, there has been a considerable interest in DNA binding properties towards different types of metal complexes. Further, many transition metal complexes have been used as tools for understanding DNA structure, as agents for mediation of DNA cleavage or as a chemotherapeutic agents. DNA is a significant cellular receptor, many chemicals bring to bear their antitumor effects by binding to DNA and by this means changes the replication of DNA and inhibits the growth of the tumor cells, which is the basis of designing new and more efficient antitumor drugs. Moreover, their effectiveness depends on the mode and affinity of their binding ability to the DNA strands [4–6]. A number of metal chelates, as agents for mediation of strand scission of duplex DNA and as chemotherapeutic agents, have been used as probes of DNA structure in solution [7–9].

8-Hydroxyquinoline (8HQ) and its derivatives have attracted unique interest for basic research and practical applications due to their therapeutic properties. 8-Hydroxyquinoline inhibits rapidly and selectively the RNA synthesis in yeast [10]. Iron bound to the lipophilic chelator 8HQ, causes substantial DNA strand breakage of cultured human lung cells [11]. Many quinoline and their derivatives have been used in the treatment of cancer, tuberculosis, diabetes, malaria, and convulsion [12–15]. In medicine particularly, a new class of drugs have been reported as potent HIV-1 inhibitors [16, 17], protein tyrosine kinase inhibitors [18], protozoal–retroviral co-infections [19], anti-HIV-1 agents [20] and therapeutic drugs for the inflammatory diseases [21]. Moreover, 8-hydroxyquinoline derivatives are also potent agents for neuro protection in Alzheimer’s, Parkinson’s, and other neuro degenerative diseases [22]. Other important applications of 8-hydroxyquinoline derivatives have been used extensively to construct highly sensitive fluorescent chemosensors for sensing and imaging of metal ions of important biological and environmental significance [23–25]. On the other hand, an excess of activated oxygen species in the forms of superoxide anion (O_2_^−^) and hydroxyl radical (OH^−^), generated by normal metabolic processes, may cause various diseases such as carcinogenesis, drug-associated toxicity, inflammation, parthenogenesis and aging in aerobic organisms [26–28]. More specifically, 8-hydroxyquinoline moiety has been mostly used for its capacity to strongly chelate metal ions, particularly Cu^+2^ and Zn^+2^ [29].

Looking to the above importance of DNA binding study of 8-hydroxyquinoline and in continuation of our earlier work [30, 31], the present paper describes microwave assisted synthesis and DNA binding interaction assay of sulfonamides substituted 8-hydroxyquinoline derivatives.

## Experimental

### Chemicals

Plasmid (pUC 19) DNA and Calf Thymus (CT) DNA were purchased from Sigma-Aldrich Chemicals Pvt. Ltd., India. All solvents and reagents were purchased commercially from Hi-Media Laboratories Pvt. Ltd., India, and were used without further purification. A solution of DNA in the buffer (Phosphate, pH 7.0) gave a ratio of UV absorbance at 260 and 280 nm of about 1.90 indicating that the DNA was sufficiently free of protein [32,33]. The CT-DNA concentrations in terms of base pair L^−1^ and in terms of nucleotide L^−1^ were determined spectrophotometrically by employing an extinction coefficient of 13,200 M^−1^ cm^−1^ (base pair) ^−1^ and 6600 M^−1^ cm^−1^ (nucleotide) ^−1^ at 260 nm, respectively [34]. Microwave experiments were carried out at atmospheric pressure in a glass vessel prolonged by a condenser, using a scientific microwave reactor (700 W Catalyst^™^ Systems, Model: Cata-R).

### Microwave assisted procedure for the synthesis of HQMABS (4-{[(8-hydroxyquinolin-5-yl)methyl]amino}benzenesulfonamide)

A mixture of 4-aminobenzenesulfonamide (0.02 mol), 5-(chloromethyl)-8-hydroxyquinoline hydrochloride (0.022 mol) and triethylamine (0.04 mol) in acetonitrile (3 mL) contained in a two-neck round bottomed flask fitted with a device condenser was heated under microwave irradiation at 350 W. After 4 minutes of microwave irradiation, the reaction was completed (Scheme 1), which was confirmed by TLC (chloroform : methanol : ammonia (70:29:1)). The reaction mixture was then poured into ice-cold water to afford off-white solid, which was filtered, washed with hot water, recrystallized from ethanol and dried under vacuum to obtain colorless crystals of HQMABS. Yield = 92.8%; mp 287 °C (identical to the product obtained in ref. [30]). MS (*m/z*) 330.0 (M+1) for C_16_H_15_N_3_O_3_S (329.4). FT-IR (KBr), ν, cm^−1^: 3401, 1401 (O–H); 3200 (N–H); 2864 (–CH_2_–), 1599 (C=N), 1144 (S=O), 1098 (C–O–H). ^1^H NMR (400 MHz, DMSO-*d*_6_, TMS): δ 9.73 (bs, 1H, OH), 8.89 (d, *J* = 8.4 Hz, 1H, H2), 7.62 (d, *J* = 8.4 Hz, 1H, H3), 8.50 (d, *J* = 8.4 Hz, 1H, H4), 7.45 (d, *J* = 8.0 Hz, 1H, H6), 7.05 (d, *J* = 8.0 Hz, 1H, H7), 7.52 (d, *J* = 8.8 Hz, 2H, H14), 6.87 (d, *J* = 8.8 Hz, 2H, H13), 6.92 (bs, 2H, SO_2_NH_2_), 6.87 (t, *J* = 5.2 Hz, 1H, C-NH), 4.66 (d, *J* = 5.2 Hz, 2H, CH_2_-N). ^13^C NMR (100 MHz, DMSO-*d*_6_, TMS): δ 153.17 (C_12_), 151.79 (C_8_), 148.36 (C_2_), 139.29 (C_10_), 133.16 (C_4_), 130.72 (C_15_), 129.61 (C_9_), 127.74 (C_14_), 127.41 (C_6_), 124.73 (C_5_), 122.26 (C_3_), 111.52 (C_13_), 110.67 (C_7_), 43.98 (C_11_).

### Microwave assisted procedure for the synthesis of the AHQMBSH (4-amino-*N′*-[(8-hydroxyquinolin-5-yl)methyl]benzenesulfonohydrazide)

A mixture of 4-acetamidobenzenesulfonyl hydrazide (0.02 mol), 5-(chloromethyl)-8-hydroxyquinoline hydrochloride (0.022 mol) and triethylamine (0.04 mol) in dry pyridine (3 mL) contained in a two-neck round bottomed flask fitted with a device condenser was heated under microwave irradiation at 350 W for 1 min. The product formation was monitored by TLC (CHCl_3_ : CH_3_OH (80 : 20)). The excess pyridine was distilled off and the residue was poured into the ice-cold water to yield a dark orange product which was filtered and washed with hot water and ethyl acetate and then dried over a vacuum desiccator. It was further hydrolyzed by treatment with solution of 10 % HCl (5 ml) and ethanol (1 ml) using microwave irradiation at 245 W for 3 min (Scheme 1). The reaction mixture was then cooled, poured into ice-cold water and neutralized with saturated NaHCO_3_ solution to yield a dark green product, which was filtered off, washed with hot-water and recrystallized from acetonitrile to afford dark green crystals, and was dried in a vacuum oven. Yield: 72%. Decomposed at 241 °C (identical to the product obtained in ref. [30]); MS (*m/z*) 344.02 (M+1) for C_16_H_16_N_4_O_3_S (344.39). FT-IR (KBr), ν, cm^−1^: 3339, 1400 (O–H); 3200 (N–H); 2964 (–CH_2_–), 1598 (C=N), 1318, 1144 (S=O), 1101 (C–O–H), 1036 (N–N). ^1^H NMR (400 MHz, DMSO-*d*_6_, TMS): δ 12.48 (s, 1H, SO_2_NH), 10.74 (s, 1H, NHC), 9.99 (bs, 1H, OH), 8.83 (d, *J* = 8.0 Hz, 1H, H2), 7.51 (d, *J* = 8.0 Hz, 1H, H3), 8.46 (d, *J* = 8.0 Hz, 1H, H4), 7.20 (d, *J* = 7.6 Hz, 1H, H6), 7.02 (d, *J* = 7.6 Hz, 1H, H7), 7.24 (d, *J* = 8.4 Hz, 2H, H14), 6.52 (d, *J* = 8.4 Hz, 2H, H13), 6.14 (bs, 2H, NH_2_), 4.87 (s, 2H, CH_2_-N). ^13^C NMR (100 MHz, DMSO-*d*_6_, TMS): δ 154.06 (C_15_), 153.09 (C_8_), 148.13 (C_2_), 138.53 (C_10_), 134.50 (C_4_), 132.47 (C_6_), 130.48 (C_13_), 128.51 (C_12_), 123.43 (C_9_), 122.01 (C_3_), 116.40 (C_5_), 112.90 (C_14_), 110.04 (C_7_), 58.27 (C_11_).

### Synthesis of complexes

**Fig. 1.**
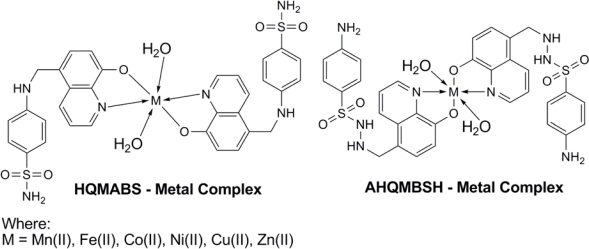
Structure of metal complexes.

Transition metal complexes of sulfonamides substituted 8-hydroxyquinoline derivatives [4-amino-*N′*-[(8-hydroxyquinolin-5-yl)methyl]benzenesulfonohydrazide (AHQMBSH) and 4-{[(8-hydroxyquinolin-5-yl)methyl]amino}benzenesulfonamide (HQMABS)] as shown in Figure 1, were synthesized by the method reported in our previous articles [30, 31].

### Methods and Measurements

The DNA-binding experiments were performed [35–37] at room temperature for ligands and its transition metal complexes. The solutions were prepared in DMSO at 10 μM concentration.

### UV–Visible experiment

UV–Visible spectra were recorded on a Shimadzu UV-Visible Spectrophotometer (Japan). UV absorption spectra were studied in the absence and in the presence of DNA (pUC 19 and Calf Thymus) at a constant concentration of the ligand and complexes (10 μM) in phosphate buffer (pH 7.0). For that, the appropriate volume of the ligand/complex (10 μl) and solution of 0–12 μl DNA (pUC 19 and Calf Thymus) were added in the phosphate buffer by maintaining the pH of the solutions at 7.0.

### Gel electrophoresis experiment

For the gel electrophoresis experiments, DNA (pUC 19 and Calf Thymus) was treated with ligand/metal complexes in phosphate buffer at pH 7.0. Agarose gel (0.8 % w/v) was prepared in TBE buffer (45 mM Tris, 45 mM boric acid and 1 mM EDTA, pH 7.3). Then 10 μl each of the incubated ligand/metal complex and DNA mixture was incubated at room temperature for 2 h. then it was loaded on the gel with tracking dye (0.25% bromophenol blue, 40% sucrose, 0.25% xylene cyanole, and 200 mM EDTA) and electrophoresis was carried out under TBE buffer system at 50 V for run. At the end of electrophoresis i.e. the end of DNA migration, the electric current was turned off. Then, the gel was stained by immersing it in water containing ethidium bromide (0.5 μg/ml) for 30–45 min at room temperature and later visualized under UV light using a transilluminator. The illuminated gel images were captured with an attached camera. Photo quantization of the gel after electrophoresis was estimated using AlphaDigiDoc^™^ RT. Version V.4.0.0 PC–Image software, California (USA).

## Results and Discussion

### UV absorption spectroscopic titration

**Fig. 2.**
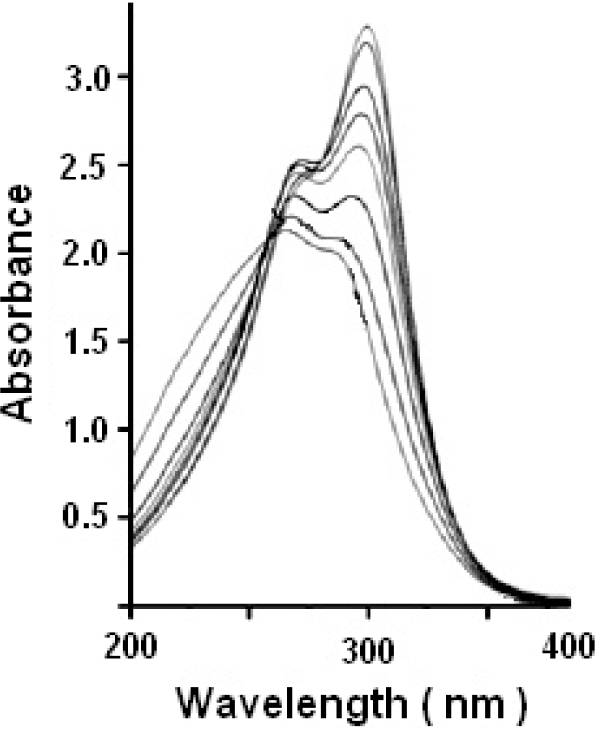
Absorption spectra of Cu-AHQMBSH complex in absence and presence of CT-DNA.

**Fig. 3.**
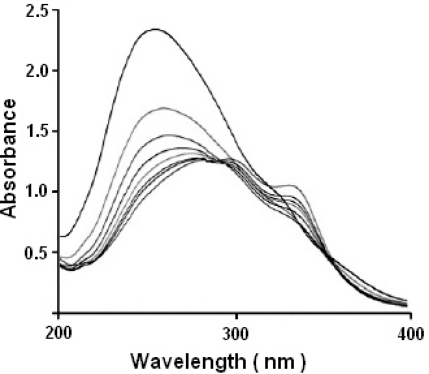
Absorption spectra of Cu-HQMABS complex in absence and presence of Plasmid DNA.

Absorption titration was carried out to monitor the interaction of a compound with DNA. The interaction of the ligands/transition metal complexes with DNA (pUC 19 and Calf Thymus) was investigated using the UV absorption spectra of complex in the absence and presence of increasing concentration of DNA and at a constant concentration of the complexes (10 μl). The observable hypochromism and red shift are usually characterized by the non-covalently intercalative binding of compound to DNA helix, due to the strong stacking interaction between the aromatic chromophore of the compound and base pairs of DNA [38, 39]. The DNA binding data of ligands/complexes were represented in Tables 1 and 2. Results of absorption titration for pUC 19 DNA as well as CT-DNA showed that no major difference was observed in the value of λ_max_. Figures 2 and 3 represent the absorption spectra of complex in the absence and presence of increasing amounts of DNA. In the UV region, the intense absorption bands were observed due to the metal complex, which is believed to be the band of intraligand transition of the coordinated groups. An addition of increasing amounts of the DNA resulted in hypochromism and bathochromic shift in the UV spectra. The hypochromism in the intraligand band reaches as high as at around 286.9 nm and 276.2 nm with red shift at the ratio of [DNA]/[Cu] in case of Cu-AHQMBSH and Cu-HQMABS respectively. These spectral characteristics suggest that the complexes had been bound to the base pairs DNA by intercalation. This bathochromism result might be due to the decrease in the energy of π→π* transition, when the π - orbital of the base pairs of DNA coupled with the π→π* orbital of the intercalated ligand.

### Gel electrophoresis

**Fig. 4.**
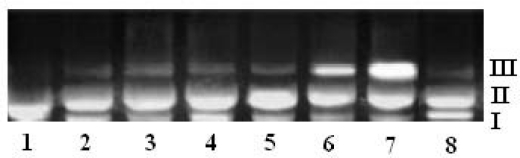
DNA binding results of ligands and metal complexes based on Gel electrophoresis.
Lane 1: Plasmid (pUC 19) DNALane 2: pUC 19 + AHQMBSHLane 3: pUC 19 + Mn-AHQMBSHLane 4: pUC 19 + Fe-AHQMBSHLane 5: pUC 19 + Co-AHQMBSHLane 6: pUC 19 + Ni-AHQMBSHLane 7: pUC 19 + Cu-AHQMBSHLane 8: pUC 19 + Zn-AHQMBSH

**Fig. 5.**
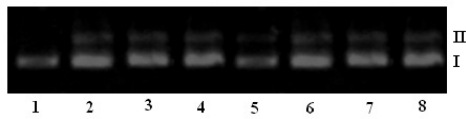
DNA binding results of ligands and metal complexes based on Gel electrophoresis.
Lane 1: Plasmid (pUC 19) DNALane 2: pUC 19 + HQMABSLane 3: pUC 19 + Mn-HQMABSLane 4: pUC 19 + Fe-HQMABSLane 5: pUC 19 + Co-HQMABSLane 6: pUC 19 + Ni-HQMABSLane 7: pUC 19 + Cu-HQMABSLane 8: pUC 19 + Zn-HQMABS

**Fig. 6.**
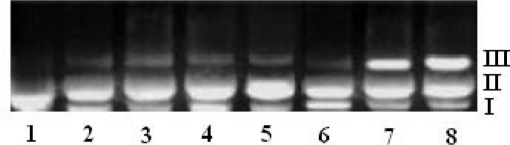
DNA binding results of ligands and metal complexes based on Gel electrophoresis.
Lane 1: CT - DNALane 2: CT - DNA + AHQMBSHLane 3: CT - DNA + Mn-AHQMBSHLane 4: CT - DNA + Fe-AHQMBSHLane 5: CT - DNA + Co-AHQMBSHLane 6: CT - DNA + Ni-AHQMBSHLane 7: CT - DNA + Cu-AHQMBSHLane 8: CT - DNA + Zn-AHQMBSH

**Fig. 7.**
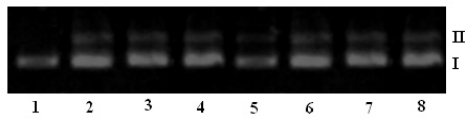
DNA binding results of ligands and metal complexes based on Gel electrophoresis.
Lane 1: CT - DNALane 2: CT - DNA + HQMABSLane 3: CT - DNA + Mn-HQMABSLane 4: CT - DNA + Fe-HQMABSLane 5: CT - DNA + Co-HQMABSLane 6: CT - DNA + Ni-HQMABSLane 7: CT - DNA + Cu-HQMABSLane 8: CT - DNA + Zn-HQMABS

The interaction of ligands/transition metal complexes with DNA was studied by electrophoresis and the results were represented in Figures 4 to 7 (Lane 3–8, are DNA + metal complex; lane 1, 2 were untreated DNA and DNA + ligand). In this study, DNA was allowed to interact with the ligands/metal complexes in presence of TAE buffer at pH 7.3 in air. When DNA was subjected to the electrophoresis after interaction and upon illumination of gel (Figures 4 to 7), the fastest migration was observed for super coiled (SC) Form I, where as the slowest migration was observed for open circular (OC) Form III and the intermediate migration was the linear (LC) Form II generated on cleavage of open circular. The intensity of untreated DNA band (Lane 1) appeared as such even after electrophoresis. Furthermore, the DNA cleavage data showed that the complexes Ni-AHQMBSH, Cu-AHQMBSH, Zn-AHQMBSH, Cu-HQMABS and Zn-HQMABS showed maximum cleavage ability compared to all the compounds (Tables 3 and 4). The variation in DNA–cleavage efficiency of ligands/transition metal complexes was due to their difference in binding ability of ligands/complexes to DNA. The intensity of lane 6, 7 and 8 were higher than others in AHQMBSH-complexes (Figures 4 and 6), which indicated that complex of Ni(II), Cu(II) and Zn(II) showed more strong binding ability with DNA. However, in case of HQMABS-complexes (Figures 5 and 7), all metal complexes showed moderate binding with DNA. The relative binding efficacy of the complexes to DNA was much higher than the ligands, and among the complexes, the binding efficacy for complexes of AHQMBSH were better than that of HQMABS-complexes. Among, the tested compounds, Cu-AHQMBSH was found to have excellent binding ability for DNA.

## Conclusion

The results of UV spectroscopy and electrophoresis measurement showed that metal complex binds with DNA via interaction as well as intercalation with the base pairs of DNA (pUC 19 and Calf Thymus). It also suggests that the covalent binding of the metal complex caused a change in the conformation of DNA such as more of intercalated and thus an increase in intensity of the band was generally observed. Moreover, the results described in this study showed that changing the ligand environment can modulate the binding property of the complex with DNA. The relative binding efficacy of the complexes to DNA was much higher than binding efficacy of ligands, especially the Cu-AHQMBSH complex has the highest binding ability to DNA, and complexes of AHQMBSH were better for DNA binding.

## Figures and Tables

**Sch. 1.**
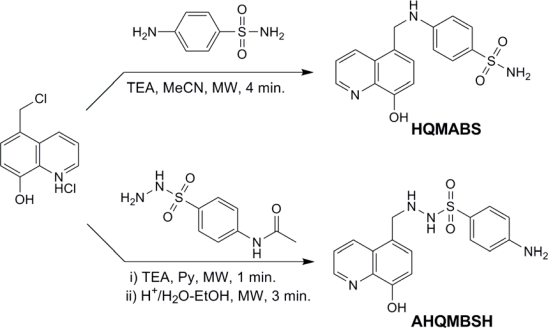
Microwave assisted synthesis of HQMABS and AHQMBSH.

**Tab. 1.** Absorption titration data of ligands and complexes with Plasmid DNA.

**Complex**	**DNA (μl)**	**Complex** **λ_max_** **(nm)**	**Ligand** **λ_max_** **(nm)**	**M(II)** **λ_max_** **(nm)**
Mn-AHQMBSH	0	282.8	263.2	271.2
	4	278.1	261.6	267.2
	8	269.4	259.4	265.6
	12	267.6	258.1	264.9
Fe-AHQMBSH	0	283.9	262.7	270.9
	4	276.2	259.8	267.6
	8	268.7	259.1	265.7
	12	267.4	258.3	264.2
Co-AHQMBSH	0	284.9	262.9	271.4
	4	282.0	260.1	268.3
	8	269.8	259.7	266.5
	12	267.5	258.4	264.6
Ni-AHQMBSH	0	280.4	262.9	270.8
	4	279.6	260.7	266.9
	8	270.1	258.7	264.7
	12	268.0	258.1	257.8
Cu-AHQMBSH	0	286.9	263.4	271.6
	4	280.2	262.0	267.9
	8	270.7	258.9	265.8
	12	268.3	257.1	263.9
Zn-AHQMBSH	0	282.1	263.4	270.9
	4	276.0	262.6	266.6
	8	261.4	259.1	265.0
	12	267.9	258.0	264.0
Mn-HQMABS	0	273.8	261.9	269.1
	4	268.6	260.5	266.0
	8	260.3	258.2	259.4
	12	258.2	257.7	257.9
Fe-HQMABS	0	274.0	261.2	268.8
	4	266.8	259.4	266.1
	8	259.7	258.2	259.2
	12	257.2	257.7	257.7
Co-HQMABS	0	275.8	262.0	268.1
	4	271.2	260.6	265.6
	8	259.4	258.3	259.4
	12	258.6	257.8	256.8
Ni-HQMABS	0	270.6	262.6	268.4
	4	269.2	260.4	266.3
	8	259.8	258.7	259.0
	12	258.1	257.3	257.8
Cu-HQMABS	0	276.2	261.7	269.2
	4	269.8	260.4	266.7
	8	263.4	258.6	259.5
	12	258.6	257.2	257.9
Zn-HQMABS	0	272.3	263.8	268.9
	4	265.6	262.1	265.8
	8	259.2	259.4	259.2
	12	257.9	255.3	257.8

**Tab. 2.** Absorption titration data of ligands and complexes with CT DNA.

**Complex**	**DNA (μl)**	**Complex** **λ_max_** **(nm)**	**Ligand** **λ_max_** **(nm)**	**M(II)** **λ_max_** **(nm)**
Mn-AHQMBSH	0	284.8	263.2	271.3
	4	279.3	261.6	267.4
	8	269.9	259.4	265.9
	12	266.8	257.9	264.2
Fe-AHQMBSH	0	285.9	262.9	271.0
	4	277.8	259.4	267.9
	8	270.7	258.7	266.5
	12	268.6	258.1	264.9
Co-AHQMBSH	0	286.2	262.9	271.9
	4	284.1	260.1	268.6
	8	270.6	259.7	266.5
	12	268.3	258.4	264.3
Ni-AHQMBSH	0	282.1	262.4	271.2
	4	280.5	260.8	267.9
	8	273.0	258.9	266.4
	12	268.7	258.0	264.6
Cu-AHQMBSH	0	286.2	263.1	271.8
	4	281.5	261.8	267.7
	8	273.3	258.6	266.3
	12	269.7	257.9	263.9
Zn-AHQMBSH	0	283.8	263.6	271.5
	4	277.2	261.3	267.1
	8	263.4	259.4	265.0
	12	258.3	257.9	264.2
Mn-HQMABS	0	272.9	262.0	268.8
	4	268.1	260.8	266.0
	8	260.0	258.2	259.1
	12	257.6	257.1	257.0
Fe-HQMABS	0	274.0	261.4	268.8
	4	266.6	259.4	266.1
	8	259.3	258.6	259.4
	12	257.2	257.7	257.7
Co-HQMABS	0	274.9	261.7	268.0
	4	271.0	260.2	265.3
	8	259.2	258.1	259.0
	12	258.5	257.6	256.9
Ni-HQMABS	0	271.2	262.8	268.5
	4	269.5	260.7	266.5
	8	259.9	258.8	259.0
	12	258.3	257.5	257.6
Cu-HQMABS	0	275.9	261.5	269.0
	4	269.4	260.1	266.3
	8	263.0	258.1	259.6
	12	258.2	257.1	257.8
Zn-HQMABS	0	271.8	263.2	268.4
	4	265.1	262.4	265.2
	8	258.7	259.7	259.0
	12	257.5	255.1	257.4

**Tab. 3.** DNA cleavage data by gel electrophoresis for AHQMBSH and their metal complexes

**Lane No.**	**Compound**	**Plasmid-DNA**			**CT-DNA**		

		**SC %**	**OC %**	**LC %**	**SC %**	**OC %**	**LC %**
1	DNA	100	–	–	100	–	–
2	AHQMBSH	41	44	15	43	46	11
3	Mn-AHQMBSH	39	46	15	39	45	16
4	Fe-AHQMBSH	45	44	11	42	43	15
5	Co-AHQMBSH	43	48	09	41	47	12
6	Ni-AHQMBSH	17	42	41	43	46	11
7	Cu-AHQMBSH	12	41	47	21	37	42
8	Zn-AHQMBSH	51	36	13	22	32	46

**Tab. 4.** DNA cleavage data by gel electrophoresis for HQMABS and their metal complexes

**Lane No.**	**Compound**	**Plasmid-DNA**			**CT-DNA**		

		**SC %**	**OC %**	**LC %**	**SC %**	**OC %**	**LC %**
1	DNA	100	–	–	100	–	–
2	HQMABS	81	19	–	78	22	–
3	Mn-HQMABS	74	26	–	76	24	–
4	Fe-HQMABS	77	23	–	71	29	–
5	Co-HQMABS	86	14	–	83	17	–
6	Ni-HQMABS	79	21	–	77	23	–
7	Cu-HQMABS	63	37	–	71	29	–
8	Zn-HQMABS	78	22	–	69	31	–
